# Effect of Local Anesthetic-Eluting Foley Catheter (FreeFoley) on Urethral Pain and Discomfort After HoLEP: A Multicenter Prospective Controlled Study

**DOI:** 10.1016/j.euros.2026.05.023

**Published:** 2026-07-02

**Authors:** Byoungkyu Han, Ki-Hyuck Moon, Jae Hoon Chung, Hwanik Kim

**Affiliations:** a Department of Urology, Perfect Urology Clinic, Seoul, the Republic of Korea; b Department of Urology, Hallym University Sacred Heart Hospital, Hallym University College of Medicine, Anyang, the Republic of Korea

**Keywords:** Benign prostatic hyperplasia, HoLEP, Analgesic-eluting urethral catheter, Bladder discomfort, Lidocaine, Prospective observational study

## Abstract

**Background and objective:**

Benign prostatic hyperplasia (BPH) is common in middle-aged and older men and often requires surgical intervention. Postoperative Foley catheterization frequently causes urethral pain and catheter-related bladder discomfort (CRBD). Systemic analgesics may be insufficient and can be contraindicated in older patients. This study evaluated the efficacy and safety of a local anesthetic-eluting Foley catheter (FreeFoley) for reducing urethral pain and discomfort after holmium laser enucleation of the prostate (HoLEP).

**Methods:**

In this prospective multicenter observational study, 108 male patients aged 50–79 yr undergoing HoLEP were allocated to either an experimental group (FreeFoley with 2% lidocaine solution, infused at 1 ml/h) or a control group (FreeFoley with normal saline, 1 ml/h). Pain and discomfort were assessed using the visual analog scale (VAS) during postoperative recovery and immediately before catheter removal. Primary outcomes were VAS scores and systemic analgesic use; secondary outcomes included catheter-related complications and patient satisfaction.

**Key findings and limitations:**

VAS scores were significantly lower in the lidocaine group than in the control group on the day of surgery (3 [2–4] vs 4 [3–5], median difference [MD], −1.1, 95% CI, −1.7 to −0.4; *p* < 0.001) and at catheter removal (2 [1–3] vs 3 [2–4], MD, −0.6, 95% CI, −1.2 to −0.2, *p* < 0.001). Analgesic use was markedly reduced (5.6% vs 28%; risk ratio [RR], 0.20, 95% CI, 0.10–0.70, *p* < 0.001). Complications occurred in 11% of patients in both groups (RR, 1.00; 95% CI, 0.36–2.78). Limitations include nonrandomization, modest sample size, and short follow-up duration.

**Conclusions and clinical implications:**

The lidocaine-eluting Foley catheter was associated with modest reductions in postoperative urethral pain, discomfort, and systemic analgesic requirements without an observed increase in complications. Further well-controlled studies are warranted to confirm the clinical benefit before routine adoption.

**  Trial registration:** Clinical Research Information Service (CRIS); KCT0011090.

## Take Home Message

The lidocaine-eluting Foley catheter was associated with modest reductions in catheter-related bladder discomfort and postoperative pain after holmium laser enucleation of the prostate without observed increases in complications. This device-based local anesthetic delivery system may provide a simple and safe approach for improving postoperative comfort and reducing systemic analgesic use.

ADVANCING PRACTICE **What does this study add?**

This prospective controlled study showed that continuous local lidocaine delivery via an anesthetic-eluting Foley catheter was associated with modest reductions in urethral pain and catheter-related bladder discomfort after holmium laser enucleation of the prostate. The intervention also reduced the need for systemic analgesics without observed increases in complications. These findings support the feasibility of device-based local analgesia as a potential adjunct to multimodal, opioid-sparing postoperative pain management. Further well-controlled studies are warranted to confirm clinical benefit and define its role in routine practice.

**Clinical Relevance**

Postoperative Foley catheterization is nearly universal after HoLEP, and catheter-related bladder discomfort remains one of the most distressing aspects of early recovery. Systemic analgesics – particularly opioids and NSAIDs – are often insufficient and carry significant risk in elderly patients with comorbidities such as cardiovascular disease, chronic kidney disease, or diabetes. The lidocaine-eluting catheter offers a simple, reproducible, and operator-independent approach to targeted local analgesia directly at the site of irritation. If confirmed in randomized controlled trials with adequate allocation concealment, this device-based strategy could reduce systemic analgesic requirements, accelerate recovery, and improve patient comfort without compromising safety. Associate Editor: Véronique Phé, M.D., Ph.D.

**Patient Summary**

We studied a new urinary catheter that slowly releases a numbing medication (lidocaine) after prostate surgery. Men who used this catheter generally experienced less pain and required fewer pain medications without more complications. This approach may help improve comfort during recovery, but more research is needed before it becomes standard treatment.

## 1 Introduction

Benign prostatic hyperplasia (BPH) is one of the most common conditions among aging men, often necessitating surgical intervention such as holmium laser enucleation of the prostate (HoLEP) [1]. Postoperative Foley catheterization, typically using 18–20-Fr catheters, is essential for hemostasis and urinary drainage but frequently results in significant urethral pain and catheter-related bladder discomfort (CRBD). CRBD is associated with reduced patient satisfaction, prolonged hospitalization, and increased systemic analgesic use [2], [3]. Conventional therapies, including opioids, nonsteroidal anti-inflammatory drugs (NSAIDs), and anticholinergics, sometimes fail to relieve symptoms sufficiently, and their systemic side effects may pose risks, especially in older patients with comorbidities [4]. To address this issue, drug-eluting urinary devices have been developed to deliver localized therapy directly to the site of discomfort. Preclinical and early clinical studies have demonstrated the feasibility of local anesthetic-eluting catheters for reducing catheter-related symptoms [5], [6], [7]. Furthermore, drug-eluting devices within the urinary tract have shown encouraging results in reducing device-related symptoms, including patient discomfort [8]. However, evidence regarding patients undergoing BPH surgery remains scarce. This study aimed to evaluate whether a lidocaine-eluting Foley catheter (FreeFoley) reduces postoperative urethral pain and discomfort compared with conventional saline-filled catheters in a multicenter prospective observational study.

## 2 Patients and methods

This multicenter prospective observational study was conducted collaboratively at Hallym University Sacred Heart Hospital and Perfect Urology Clinic between June 2025 and September 2025. The study protocol was reviewed and approved by the Institutional Review Board (IRB no. HALLYM 2025-03-015-001), and all participants provided written informed consent prior to enrollment. The study was conducted in accordance with the Declaration of Helsinki and relevant ethical guidelines.

### 2.1 Participants

Male patients aged 50–79 yr who were diagnosed with BPH and scheduled to undergo HoLEP were eligible for inclusion. Exclusion criteria included a history or current diagnosis of prostate or bladder cancer, urethral stricture, bladder pain syndrome, known hypersensitivity or contraindications to lidocaine (eg, allergy to amide-type local anesthetics or severe cardiac conduction disturbances), contraindications to anticoagulation therapy, neurogenic bladder dysfunction, severe renal or hepatic impairment, and unwillingness to participate.

### 2.2 Sample size calculation

Prior to study initiation, the sample size was calculated based on the primary endpoint of the difference in urethral pain and discomfort measured using the visual analog scale (VAS). To detect a clinically significant difference of 1.5 points in median VAS scores, assuming a standard deviation of 2, a statistical power of 90%, and a 2-sided α level of 0.05, a total of 76 patients were required. Considering an anticipated dropout rate of 30%, 108 patients were enrolled, with 54 patients allocated to each group.

### 2.3 Allocation and blinding

Participants were allocated in a 1:1 ratio to either the experimental group, which received the local anesthetic-eluting Foley catheter (FreeFoley) infused with 2% lidocaine solution at a rate of 1 ml/h, or the control group, which received a FreeFoley catheter infused with normal saline at the same rate. Group allocation was performed using a predefined alternating sequence. Although allocation concealment was not feasible due to the alternating sequence design, both patients and outcome assessors were blinded to group assignment to minimize performance and detection bias. To further maintain blinding integrity, the lidocaine and saline solutions were prepared by a study nurse who was not involved in outcome assessment, and the infusion reservoirs were visually identical. Surgeons were not involved in postoperative pain assessment, and outcome evaluators remained blinded to group allocation throughout data collection and analysis.

### 2.4 Intervention details

All HoLEP procedures were performed by experienced board-certified urologists, each of whom had performed more than 500 HoLEP cases before study initiation. Collectively, the participating surgeons had performed more than 5000 HoLEP procedures. Because these surgeons had surpassed the learning curve and independently managed both outpatient care and surgical procedures, surgeon-specific variability was considered unlikely to affect the results. HoLEP was performed using a holmium:yttrium-aluminum-garnet laser with standardized energy settings (pulse energy, 2 J; frequency, 40 Hz) according to institutional protocols. The FreeFoley catheter in the experimental group was prepared by infusing a total volume of 30 ml, comprising 15 ml of 2% lidocaine and 15 ml of normal saline, into the catheter reservoir to achieve continuous elution at approximately 1 ml/h postoperatively. The control group catheter contained 30 ml of normal saline with the same infusion rate. All patients underwent standard postoperative catheter management according to institutional protocols. The Foley catheter size (18 Fr or 20 Fr) was determined by the operating surgeon based on hemostasis. Because all participants successfully accommodated a 26-Fr resectoscope without prior urethral dilation, the urethral caliber was considered sufficient for both 18-Fr and 20-Fr catheters. The 0.67 mm difference in diameter between these two sizes was considered clinically insignificant for urethral irritation. Statistical analysis confirmed no significant difference in catheter size distribution between the two groups (*p* = 0.6), thereby minimizing catheter caliber as a potential confounding factor for postoperative discomfort.

### 2.5 Outcome measures

The primary outcome was the degree of urethral pain and bladder discomfort, quantified using the VAS at two time points: immediately after postoperative recovery and immediately before catheter removal. CRBD was defined as suprapubic discomfort, urgency, or a burning sensation attributed to the indwelling Foley catheter. Urethral pain and discomfort were quantified using a 10-point VAS, with higher scores indicating greater symptom severity. Secondary outcomes included systemic analgesic use during the postoperative period; catheter-related complications, such as hematuria, urinary tract infection, and urinary retention; and patient-reported satisfaction and recommendation scores measured via standardized questionnaires. Systemic analgesic use was recorded as the administration of any parenteral nonsteroidal anti-inflammatory drug or opioid during the postoperative catheterization period. Specifically, patients were asked, “Would you recommend this procedure to your acquaintances or family members?” Response options were as follows: 1, “No, I would not recommend it”; 2, “Not sure”; 3, “Yes, I would recommend it.”

### 2.6 Safety monitoring

All adverse events and catheter-related complications were monitored throughout hospitalization. Serious adverse events were promptly reported to the institutional review boards, and their causality with the intervention was assessed by independent investigators.

### 2.7 Statistical analysis

Continuous variables were summarized as median (interquartile range [IQR]) and compared using the Mann–Whitney U test. Categorical variables are presented as frequency (percentage) and were compared using the chi-square test; Fisher exact test was used only when expected cell counts were 0 or 1. Effect estimates are presented as between-group median differences for continuous outcomes and RRs for binary outcomes, each with corresponding 95% confidence intervals (95% CIs). Between-group median differences and their 95% CIs were estimated using a nonparametric bootstrap approach with 1000 resamples. All statistical tests were 2-sided, and *p* < 0.05 was considered statistically significant. Data analyses were performed using IBM SPSS Statistics, version 27.0 (IBM Corp., Armonk, NY).

## 3 Results

A total of 108 patients with BPH undergoing HoLEP were analyzed in two groups without censoring. None of the patients met the exclusion criteria, including contraindications to lidocaine. In total, 54 patients received an indwelling Foley catheter continuously infused with 2% lidocaine solution (experimental group), and 54 received an indwelling Foley catheter infused with normal saline (control group). All patients underwent spinal anesthesia, and Foley catheter size ranged from 18 to 20 Fr. Catheter size distribution did not differ significantly between groups (*p* = 0.6).

There was no evidence of between-group differences in baseline demographic and clinical characteristics (Table 1). Specifically, no differences were observed in age (72 [65–79] vs 72 [64–80] yr; *p* = 0.9), total prostate size (71 [52–101] vs 69 [50–103] ml; *p* = 0.7), transition zone volume (37 [20–61] vs 36 [18–64] ml; *p* = 0.8), serum prostate-specific antigen (PSA) level (4.5 [1.8–9.4] vs 4.9 [2.0–9.0] ng/ml; *p* = 0.7), International Prostate Symptom Score (IPSS) total score (*p* = 0.4), IPSS quality of life score (*p* = 0.6), resected prostate specimen weight (*p* = 0.3), total operative time (*p* = 0.8), prevalence of diabetes (*p* = 0.6), prevalence of hypertension (*p* = 0.7), or total delivered energy (*p* = 0.5).

**Table 1:** Baseline characteristics of the study population

Variable	Experimental group (*n* = 54)	Control Group (*n* = 54)	*p*-value
Age, yr	72 (65–79)	72 (64–80)	0.9
Prostate size, ml	71 (52–101)	69 (50–103)	0.7
Transition zone volume, ml	37 (20–61)	36 (18–64)	0.8
PSA level, ng/ml	4.5 (1.8–9.4)	4.9 (2.0–9.0)	0.7
IPSS total score	21 (15–27)	22 (16–28)	0.4
QoL score	4 (3–5)	4 (3–5)	0.6
Resected specimen weight, ml	61 (40–103)	53 (38–91)	0.3
Operative time, min	58 (40–86)	56 (38–82)	0.8
Diabetes mellitus, *n* (%)	10 (19%)	13 (24%)	0.6
Hypertension, *n* (%)	25 (46%)	22 (41%)	0.7
Total delivered energy, kJ	68 (48–88)	66 (50–84)	0.5

Continuous variables are presented as median (interquartile range), and categorical variables are presented as frequency (percentage).

IPSS = International Prostate Symptom Score; PSA = prostate-specific antigen; QoL = quality of life.

Regarding the primary outcome, the experimental group had significantly lower urethral pain and CRBD than the control group. On the day of surgery (postoperative day 0), the median VAS score for discomfort was 3 (2–4) in the experimental group, significantly lower than 4 (3–5) observed in the control group (median difference [MD], −1.1; 95% CI, −1.7 to −0.4, *p* < 0.001). At catheter removal (postoperative day 1), the experimental group had reduced pain and discomfort, with a median VAS score of 2 (1–3), compared with 3 (2–4) in the control group (MD, −0.6; 95% CI, −1.2 to −0.2; *p* < 0.001).

In addition to subjective pain assessment, objective evaluation of analgesic use showed a markedly reduced need for additional systemic pain medication in the experimental group. Only three patients (5.6%) in the lidocaine group required parenteral diclofenac compared with 15 patients (28%) in the control group (RR, 0.20; 95% CI, 0.10–0.70; *p* < 0.001). Although opioid analgesics (tramadol) were administered to four patients in the control group and none in the experimental group, this difference did not reach statistical significance (risk difference, −7%; 95% CI, −14% to 0%; *p* = 0.12). No patients required ketorolac or pethidine in either group.

No evidence of differences was observed in secondary measures evaluating patient satisfaction and willingness to recommend the treatment. Median satisfaction scores were 4 (3–5) in the experimental group and 4 (3–5) in the control group (MD, 0; 95% CI, −1 to 1; *p* = 0.5). Similarly, recommendation scores were nearly identical between groups (3 [2–3] vs 3 [2–3]; MD, 0; 95% CI, −1 to 1; *p* = 0.8). The distribution of recommendation levels (levels 2 and 3) did not differ between groups (Table 2).

**Table 2:** Postoperative outcomes between the two groups

Variable	Experimental group (*n* = 54)	Control group (*n* = 54)	Effect estimate, *p*-Value
VAS score on operation day	3 (2–4)	4 (3–5)	**MD, −1.1 (95% CI, −1.7 to −0.4); *p*** < **0.001**
VAS score on POD 1	2 (1–3)	3 (2–4)	**MD, −0.6 (95% CI, −1.2 to −0.2); *p*** < **0.001**
Patient satisfaction score	4 (3–5)	4 (3–5)	MD, 0 (95% CI, −1 to 1); *p* = 0.5
Recommendation score	3 (2–3)	3 (2–3)	MD, 0 (95% CI, −1 to 1); *p* = 0.8
Recommend level 2 (Not Sure), *n* (%)	17 (31)	18 (33)	RD, −2% (95% CI, −17% to 13%); *p* = 1
Recommend level 3 (Yes, I would recommend it), *n* (%)	37 (69)	36 (67)	RD, 2% (95% CI, −13% to 17%); *p* = 1
Diclofenac use, *n* (%)	3 (5.6)	15 (28)	**RR, 0.20 (95% CI, 0.10–0.70); *p*** < **0.001**
Tramadol use, *n* (%)	0 (0)	4 (7.4)	RD, −7% (95% CI, −14% to 0%); *p* = 0.12
Ketorolac use, *n* (%)	0 (0)	0 (0)	–
Pethidine use, *n* (%)	0 (0)	0 (0)	–

Continuous variables are presented as median (interquartile range), and categorical variables are presented as frequency (percentage).

CI = confidence interval; MD = median difference; POD = postoperative day; RD = risk difference; RR = risk ratio; VAS = visual analog scale.

The incidence of catheter-associated complications, including hematuria, urinary tract infection, and acute urinary retention, was 6 of 54 patients (11%) in both groups (risk difference, 0%; 95% CI, −12% to 12%; RR, 1.00; 95% CI, 0.36–2.78; *p* = 1), suggesting that continuous local lidocaine infusion via the catheter did not increase adverse events or compromise patient safety. During the 3-mo follow-up period, no catheter-related complications of Clavien–Dindo grade III or higher were observed. Minor complications included mild urethral pain requiring conservative management (experimental group, 1 patient; control group, 2 patients), prolonged urethral bleeding lasting more than 1 mo (experimental group, 1 patient; control group, 1 patient), simple urinary tract infection (experimental group, 1 patient; control group, 1 patient), and prolonged dysuria persisting beyond 1 mo (experimental group: 3 patients; control group, 2 patients). Major catheter-related complications, such as meatal stenosis and urethral stricture, were not observed during the follow-up period in either group. No cases of acute urinary retention or capsular perforation occurred in either group.

## 4 Discussion

This prospective controlled study showed that the use of a lidocaine-eluting Foley catheter was associated with reduced CRBD and decreased systemic analgesic use in patients undergoing surgery for BPH. These findings are clinically meaningful, given that postoperative Foley catheterization is nearly universal after HoLEP, and CRBD remains one of the most distressing complications during the early recovery period [3], [9]. By delivering local anesthetic directly to the site of irritation, the FreeFoley catheter may provide targeted symptom relief that is difficult to achieve with systemic medications alone. Importantly, there was no evidence of a difference in total delivered laser energy between the two groups (experimental group, 68 (48–88) vs control group: 66 (50–84) kJ, *p* = 0.5), suggesting that the observed reduction in postoperative pain was unlikely to be attributable to differences in surgical energy application. The absence of significant between-group differences in complication rates or patient satisfaction further supports the short-term safety of this approach. Collectively, these findings suggest that a local anesthetic-eluting catheter may improve patient comfort during the postoperative recovery period without compromising surgical safety [5].

One of the most clinically relevant findings of this study was the reduction in CRBD observed in patients receiving the lidocaine-eluting Foley catheter compared with controls. This finding is consistent with previous studies demonstrating that localized delivery of anesthetic agents can modulate nociceptive pathways in the bladder and urethra, where systemic analgesics may fail to achieve adequate concentrations [2]. Previous studies evaluating topical gels or ointments reported limited and transient effects, largely because of rapid dilution or washout within the urinary tract. In contrast, the continuous infusion system of the FreeFoley catheter provides sustained exposure of the urethral mucosa to lidocaine, thereby maintaining therapeutic concentrations over time [4]. This mechanistic advantage may explain the reduction in VAS scores observed in the present study. Importantly, our findings suggest that this benefit was achieved without an observed increase in adverse events, supporting both the potential efficacy and safety of this novel device. From a translational perspective, this technology may help address the need for improved perioperative analgesic strategies in patients undergoing BPH surgery [6], [7].

Another important consideration is the reduced use of systemic analgesics. In our cohort, fewer patients in the intervention group required parenteral analgesics. This finding has broader implications, particularly for older patients who undergo BPH surgery and often have comorbidities such as cardiovascular disease, diabetes, or chronic kidney disease. The use of opioids or nonsteroidal anti-inflammatory drugs in this population is limited by the risks of respiratory depression, delirium, gastrointestinal bleeding, and renal impairment [10]. By mitigating catheter-related pain at its source, the FreeFoley catheter may reduce reliance on systemic medications and their associated risks. In addition, lower analgesic use may accelerate postoperative recovery, facilitate earlier mobilization, shorten hospital stay, and ultimately improve the cost-effectiveness of surgical care [11], [12], [13]. These broader health economic outcomes were not the primary focus of the present study but represent important avenues for future research.

This study also contributes to the growing body of evidence supporting device-based analgesic strategies in urology. Drug-eluting stents, for example, have shown promise in reducing flank pain and urinary urgency associated with ureteral stent placement [14], while experimental studies of nanofiber-coated stents demonstrated attenuation of granulation tissue and improved biocompatibility [15]. The FreeFoley catheter represents a parallel innovation tailored to the unique challenges of postoperative bladder drainage. Continuous intraluminal lidocaine delivery did not result in any lidocaine-related adverse events, including local irritation, systemic toxicity, or allergic reactions, supporting the short-term safety of this approach. In addition, the incidence of catheter-related complications, including hematuria, urinary tract infection, urinary retention, and urethral injury, was comparable between groups. By focusing on patients undergoing HoLEP, for whom catheter retention is standard practice, we were able to delineate the device’s role in symptom control. Nonetheless, some limitations merit emphasis. The relatively short follow-up period precluded assessment of long-term outcomes such as delayed infections, urethral stricture formation, or chronic pain syndromes. Additionally, the study population consisted exclusively of male patients with BPH; whether similar benefits would be observed in female patients or in other surgical settings remains unknown. One randomized controlled trial (RCT) of 110 patients evaluated bladder spasm and CRBD after pudendal nerve block followed by HoLEP [16]. They reported that a bilateral pudendal nerve block at the entrance of the pudendal canal resulted in a lower incidence of CRBD and bladder spasm, less postoperative pain, and reduced analgesic use. This finding supports the concept that regional or local anesthetic strategies targeting afferent neural pathways can provide symptom control comparable to that achieved with systemic analgesics. Notably, the mechanism of action differs from that of local anesthetic-eluting catheters, which act directly on the urethral mucosa and bladder neck rather than by blocking peripheral nerves. Although peripheral nerve blocks can be technically challenging and may require ultrasound guidance by anesthesiologists, an anesthetic-eluting Foley catheter offers a simpler, reproducible, and less operator-dependent technique that can be applied broadly in patients requiring postoperative catheterization. Larger multicenter trials with diverse populations and standardized protocols are needed to validate these findings.

Despite its strengths, this study has several limitations. First, the sample size was relatively small and may not have provided sufficient statistical power to detect smaller differences in secondary outcomes, such as patient satisfaction or rare catheter-related complications. Although between-group differences in VAS scores were statistically significant at the prespecified time points, the median differences were generally below the prespecified minimal clinically important difference of 1.5 points. Therefore, the average symptomatic benefit should be considered modest and requires confirmation in well-controlled studies. Second, the follow-up duration was short and limited to the immediate postoperative period until catheter removal; therefore, long-term outcomes such as late-onset infections, urethral stricture formation, or chronic pain syndromes could not be evaluated. Third, the study population consisted exclusively of male patients undergoing HoLEP, which may limit the generalizability of the findings to other patient populations or surgical settings. In addition, urethral calibration was not standardized, and catheter size selection was individualized according to intraoperative findings and surgeon preference. This lack of standardization might have influenced postoperative discomfort and represents an important limitation of the present study. Future studies using standardized urethral calibration protocols are warranted. Fourth, although this study was conducted prospectively with blinded outcome assessment, group allocation was performed using an alternating sequence rather than true randomization. This observational design precluded allocation concealment and may have introduced selection bias. Therefore, the findings should be interpreted cautiously, and confirmation in fully randomized controlled trials with adequate allocation concealment is warranted. Finally, because this was a single-country multicenter study, cultural and healthcare system-related factors may have influenced patient-reported outcomes. External validation in other populations is therefore warranted.

## 5 Conclusion

The local anesthetic-eluting Foley catheter (FreeFoley) was associated with reduced urethral pain, catheter-related bladder discomfort, and systemic analgesic use following BPH surgery, without increasing catheter-related complications. Nevertheless, given the prospective observational study design, these findings require confirmation in well-designed randomized controlled trials with adequate allocation concealment.

  ***Author contributions:*** Hwanik Kim had full access to all the data in the study and takes responsibility for the integrity of the data and the accuracy of the data analysis.

  *Study concept and design*: Han, Kim.

*Acquisition of data*: Han, Ki- Moon, Kim.

*Analysis and interpretation of data*: Han, Kim.

*Drafting of the manuscript*: Han and Kim.

*Critical revision of the manuscript for important intellectual content*: Han, Moon, Chung, Kim.

*Statistical analysis*: Han, Moon, Chung, Kim.

*Obtaining funding*: None.

*Administrative, technical, or material support*: Han, Kim.

*Supervision*: Han, Kim.

*Other* (specify): None.

  ***Financial disclosures:*** Hwanik Kim certifies that all conflicts of interest, including specific financial interests and relationships and affiliations relevant to the subject matter or materials discussed in the manuscript (eg, employment/affiliation, grants or funding, consultancies, honoraria, stock ownership or options, expert testimony, royalties, or patents filed, received, or pending), are the following: None.

  ***Funding/Support and role of the sponsor*:** None.
